# Effects of Relocation and Individual and Environmental Factors on the Long-Term Stress Levels in Captive Chimpanzees (*Pan troglodytes*): Monitoring Hair Cortisol and Behaviors

**DOI:** 10.1371/journal.pone.0160029

**Published:** 2016-07-27

**Authors:** Yumi Yamanashi, Migaku Teramoto, Naruki Morimura, Satoshi Hirata, Miho Inoue-Murayama, Gen'ichi Idani

**Affiliations:** Wildlife Research Center, Kyoto University, Kyoto, Japan; Georgia State University, UNITED STATES

## Abstract

Understanding the factors associated with the long-term stress levels of captive animals is important from the view of animal welfare. In this study, we investigated the effects of relocation in addition to individual and environmental factors related to social management on long-term stress level in group-living captive chimpanzees by examining behaviors and hair cortisol (HC). Specifically, we conducted two studies. The first compared changes in HC levels before and after the relocation of 8 chimpanzees (Study 1) and the second examined the relationship between individual and environmental factors and individual HC levels in 58 chimpanzees living in Kumamoto Sanctuary (KS), Kyoto University (Study 2). We hypothesized that relocation, social situation, sex, and early rearing conditions, would affect the HC levels of captive chimpanzees. We cut arm hair from chimpanzees and extracted and assayed cortisol with an enzyme immunoassay. Aggressive behaviors were recorded ad libitum by keepers using a daily behavior monitoring sheet developed for this study. The results of Study 1 indicate that HC levels increased during the first year after relocation to the new environment and then decreased during the second year. We observed individual differences in reactions to relocation and hypothesized that social factors may mediate these changes. In Study 2, we found that the standardized rate of receiving aggression, rearing history, sex, and group formation had a significant influence on mean HC levels. Relocation status was not a significant factor, but mean HC level was positively correlated with the rate of receiving aggression. Mean HC levels were higher in males than in females, and the association between aggressive interactions and HC levels differed by sex. These results suggest that, although relocation can affect long-term stress level, individuals’ experiences of aggression and sex may be more important contributors to long-term stress than relocation alone.

## Introduction

Social housing is essential to the welfare of some captive animals e.g. [1–3]. Wild chimpanzees form multi-male and multi-female groups [4–6], and evidence suggests that chimpanzees who are separated from their conspecifics show diverse abnormal behaviors, including self-injurious and stereotypic behaviors, and fail to show certain appropriate, normal social and reproductive behaviors e.g. [7–11]. Therefore, providing social stimulation in the form of interaction with conspecifics is one of the most important parts of the care of captive chimpanzees. However, social management is one of the most difficult parts of captive management [2,12,13]. Although the formation of complex social groups comparable to that of wild groups is recommended, it can sometimes result in negative consequences. For example, male chimpanzees are aggressive in nature [14]; keeping several adult males in a captive environment with females often results in escalated aggression. As a result, surplus males emerge, and unnatural social compositions, such as all-male groups, are sometimes formed to solve the problem of surplus animals [15]. In addition, only adolescent females normally migrate from their natal groups in the wild, whereas both females and males in captivity are relocated between institutions—on artificial schedules—due to breeding programs, retirement from research, closure of institutions, etc. [16,17] and integrated into new social groups. These factors may lead to long-term stress in captive chimpanzees. Furthermore, in human and non-human animals, social interaction can have both positive and negative consequences, depending on the circumstances and the quality of the relationship e.g. [18,19]. As social relationships are unpredictable and can affect animal welfare for over long period of time, social management plays a role in maximizing the positive effects of these social relationships while reducing the stress derived from them.

Understanding the factors associated with long-term stress is particularly important, as it can have profound effects on animal welfare. Glucocorticoids (GCs), steroid hormones secreted by the adrenal glands of vertebrates, are frequently used as an indicator of physiological and psychological stress, because they often increased when organisms face stressors [20,21]. GCs generally increase available energy, and their acute increases are generally considered adaptive in stressful situations. However, prolonged exposure to GCs can result in a number of maladaptive consequences, such as neuronal cell death, insulin resistance, muscle and bone atrophy, poor wound healing, hypertension, and even collapse of the immune system to the point of death [22]. Prolonged exposure can also affect their reproduction [23] and accelerate or increase the likelihood of acquiring certain illnesses, such as cardiovascular disease, which is one of the common causes of death in captive great apes [20,23–26]. Cortisol is the primary GC released in primates and has historically been measured in the blood, saliva, urine, and feces of many primate species e.g. [27–35]. Although these samples offer advantages in the measurement of relatively short-term stress, they are difficult to use to monitor long-term stress. Bahr et al. [36] compared the temporal excretion of cortisol metabolites in the urine and feces of a chimpanzee. They reported that the peak of excretion in urine was found 4.8 h after labeled cortisol was administered intravenously; in feces, it was recovered 22.2 h after administration. Even fecal samples can change within 24 h of cortisol secretion, rendering repeated sampling necessary. Additionally, the cortisol concentration often changes with storage method and duration [37,38] and can also be affected by diurnal rhythms [39]. Such issues make it difficult to investigate long-term stress in captive wild animals.

Hair cortisol (HC) has been shown to be a useful measure of long-term hypothalamic–pituitary–adrenal (HPA) axis activation in several species including humans [40–48] and has attracted attention as a way to overcome the aforementioned problems. Substances from blood are absorbed by growing hair follicles and accumulate as hair grows [49]. Therefore, HC can reflect the accumulation of cortisol in the hair shaft over several months.It was reported that HC levels increase after relocation [41] and are associated with behavioral phonotype [50,51], population density [52], and ecological disturbance caused by humans [46,53]. Recently, we developed an HC assay to monitor cortisol accumulation for several months in captive chimpanzees and found a link between HC levels and aggression [54]. Although body regions can affect the HC analysis [54–56], we found a similar trend in the change in HC levels between the proximal and distal parts of hair segments obtained from different body regions [57]. Hair color and other methodological factors can also influence the HC results, but HC is stable over time [57,58] and is advantageous for long-term monitoring of cortisol in various captive settings, provided these methodological pitfalls are avoided. This new methodology enables the long-term stress levels of captive chimpanzees to be assessed more efficiently. However, few studies have used this novel technique to assess the effects of the husbandry and management of captive animals.

Our goal was to investigate the relationship between factors relevant to social management and long-term stress levels in captive chimpanzees via two specific approaches. First, we investigated the effects of the relocation and acclimation of chimpanzees by monitoring behaviors and HC in a group of chimpanzees over a 3-year period. We first focused on relocation as it is a challenging event that requires animals to adapt to a new physical and social environment and thus requires particular care and consideration from the animal caretakers. Understanding how relocation affects long-term stress is useful for creating strategies to mitigate the stress derived from relocation and to maintain the physical and mental health of the animals. However, despite the fact that chimpanzees are one of the most common great ape species in captivity and that the animals are frequently relocated between institutions, little is known about the relationship between long-term stress and the relocation of chimpanzees living in social housing [59–61]. Previously, Schapiro et al. [16] reported the effects of the relocation of 72 chimpanzees on physiological parameters obtained from blood. They found that hematological, clinical chemistry, and immunological parameters changed significantly immediately after relocation and reported that the immunological parameters of some of the chimpanzees had not returned to their baseline levels 12 weeks after relocation, indicating that chimpanzees may need long periods of acclimation after being moved to a new environment. Another chimpanzee study suggested that the effects of relocation depend on rearing history [62]. Reimers et al. [62] measured changes in fecal GC metabolites after the transportation and resocialization of ex-laboratory chimpanzees and found that chimpanzees who had experienced early deprivation showed more prolonged cortisol increases than those who had experienced late deprivation. Recent studies in rhesus macaques found similar results. Devenport et al. [41] reported that the HC levels of rhesus macaques increased 14 weeks after relocation and decreased 35 weeks after relocation. Furthermore, Dettmer and Novak [63] suggested that mother-reared rhesus macaques showed a weaker response to relocation. Therefore, relocation can influence many welfare indicators for the long-term and rearing history, and other factors, such as age, sex, group formation, and aggressive interactions, may modify stress reactions to relocation. We hypothesized that relocation can lead to long-term stress in captive chimpanzees. Specifically, we predicted that the HC level of chimpanzees would increase after relocation and decrease 1 year after relocation, as observed in Davenport et al. (40). We also predicted that there might be sex differences in the response to relocation, because female wild chimpanzees migrate into other social groups after puberty, whereas male chimpanzees do not [4].

Second, we investigated the factors underlying individual differences in HC levels among 58 captive chimpanzees. As noted above, there can be individual differences in reactions to relocation, and this might be important when considering a specific strategy to mitigate the long-term stress of chimpanzees. However, it is difficult to test these hypotheses with the first approach mentioned above due to the impracticality of obtaining sufficient numbers of relocated chimpanzees. Additionally, various individual (e.g., age, sex, and early-rearing conditions) and environmental factors can influence the HC levels at the same time. Therefore, we focused on individual differences in HC levels and investigate the factors influencing the average HC levels of captive chimpanzees using statistical approach sometimes used in the previous studies of animal welfare. For example, Shepherdson et al. [64] compared individual fecal GC metabolites (FGM) of 55 polar bears living in 20 zoological institutions to investigate individual and facility level factors affecting FGM levels. They found that temperament, stereotypic behaviors and some facility characteristics were related to FGM levels. This approach based on epidemiological studies has proven to be a powerful tool for elucidating the risk factors that decrease animal welfare [64,65]. In this regard, we test four specific hypotheses. First, we hypothesized that relocation can influence average HC levels. We predicted that immigrant or relocated chimpanzees who came from other institutions would have higher HC levels than native chimpanzees who did not experience changes in their physical and social environment. Second, we hypothesized that social situation can influence the HC responses. Previously, we found a positive correlation between HC and the rate of receiving aggression, whereas the rate of initiating aggression was not related to HC levels in six male chimpanzees [54]. We predict that similar results would be obtained with a larger sample size, but, as males and females have different social characteristics, we hypothesized that the relationship between HC and aggression might be sex-dependent. We also predicted that social composition influences HC levels. As noted previously, all-male groups sometimes form to solve the problem of surplus male chimpanzees [12,13], and such social groups might constitute a feasible management strategy for captive chimpanzees. However, no study has investigated differences in stress responses between the males in all-male groups and those in mixed-sex groups males, despite the fact that all-male group formation is not observed in wild chimpanzees, which differs from the case in gorillas [66]. If all-male group formation poses excessive physiological challenges, the HC levels could be higher in individuals living in such groups. Third, early-rearing experience can also influence HC levels. Studies have shown that animals who have experienced maternal separation early in life have altered HPA responses e.g. [67–69]. Because impaired social abilities were observed in individuals who were separated from their mother early in life e.g. [11,70], such individuals might be suffering from such a deficiency. In that case, we would expect them to have higher HC levels than other individuals.

## Materials and Methods

### Subjects

The subjects were 58 chimpanzees (35 males, 23 females; age range: 5–44 years) living in Kumamoto Sanctuary (KS), Kyoto University, Japan. Eight were relocated from the Great Ape Research Institute (GARI) in January 2013. Established in 2007, KS was the first chimpanzee sanctuary in Japan (it was renamed from Chimpanzee Sanctuary Uto (CSU) in 2011 when the institution was passed onto Kyoto University from Sanwa Kagaku Research Institute. For more information see [12]. KS accommodates ex-laboratory chimpanzees and chimpanzees considered surplus in Japanese zoos. KS accepted 15 chimpanzees from other institutions between 2008 and 2012. It promotes the social life of chimpanzees, and three types of social groups have formed: all-male groups; one-male and multi-female groups; and multi-male, multi-female groups. The members of the all-male groups are changed periodically to provide social stimulation and prevent escalated aggression, directed especially toward immigrant individuals. Decisions regarding social group formation were also based on the chimpanzees’ choices. The chimpanzees sometimes refused to move or moved to near the door connected to different enclosures voluntarily. Such behaviors were considered when making the decisions. All individuals had access to both indoor and outdoor enclosures, most of which are cage style (*i*.*e*., a building with a roof), although one outdoor enclosure (about 270 m^2^ in area) has no roof. The outdoor cages range in size from about 70 m^2^ in area and 5.4 m in height to about 120 m^2^ in area and 12 m in height. All these outdoor cages are connected to other cages. Most of the chimpanzees used multiple cages, and some of them used different enclosures from day to day. Passages totaling 150 m in length that connect several cages within KS were introduced, and groups of chimpanzees could access the passages in turn for exploration. They had free access to water at any time, and regular meals (consisting mainly of fruits, vegetables, and monkey pellets) were provided three times per day. Additionally, routine feeding enrichment (e.g., juice feeders, puzzle feeders, browsing opportunities, and foods concealed in boxes or newspapers) were changed daily. Other types of environmental enrichment were also provided. For example, fire hoses, ropes, hammocks, climbing structures, and substrate materials were installed, and natural vegetation was planted to increase the complexity of the physical environment. Spaces were also available for the chimpanzees to escape from rain, strong sunlight, and cold, and they were provided with comfortable bedding materials for day- and night-time sleep. Materials that they could manipulate freely were also provided, such as toys, buoys, and sacks. For additional details on environmental enrichment, please see [71]. In addition to the lifelong care of these chimpanzees and bonobos, non-invasive research (cognitive, behavioral, endocrinological, and genetic) is conducted at KS.

## General Methods

### Hair cortisol (HC) assessment

Basic methods to quantify HC levels were based on our previous study [54]. Briefly, samples were collected from chimpanzees by cutting arm hair with scissors. Samples were collected from similar parts of the arms. Samples were cut with scissors by keepers or researchers at each institute (KS: MT, NM, SH, and other keepers; GARI: keepers) who were familiar with the individual animals. We were able to obtain hair samples from all chimpanzees. There was some small variation in the sampling procedures across individuals. Hair samples were collected by asking the subject chimpanzees to show their arm parts through the cage mesh or to extend their arms through a small window in their home cages or enclosures, or when people were together with the subject chimpanzees in the same room. In two cases, we collected hair samples when chimpanzees were anesthetized for their health check. Although we tried to cut the hair at the skin surface in all the cases, about 5 mm of hair was often left. To check whether we were able to obtain stable results with this methodology, we compared the HC levels obtained from similar body parts on the same day in our previous study [57]. We found that the samples were significantly correlated and the absolute values did not differ significantly. Therefore, although small intra- and inter-individual variation exists in terms of hair sample collection, the previous study showed that the procedures can produce consistent results [57]. We also collected multiple hair samples from each individual to use average HC levels for comparisons, to minimize the effects of such sampling errors and possible inter-individual variation in the hair growth rate. Samples were stored at ambient temperature until analysis. Samples were washed with 5 mL isopropanol and dried. Samples were then ground into a fine powder using a Precellys 24 tissue homogenizer (Bertin Technologies, Orléans, France). The powdered samples were weighed and placed in 2-mL tubes, and 1-mL methanol was added. Cortisol was extracted by shaking the tubes for 24 h at ambient temperature. Following extraction, the samples were centrifuged, and 0.6 mL of supernatant was aliquoted into different tubes and evaporated by vacuum oven at 80°C. Samples were reconstituted using phosphate buffer, and cortisol concentrations were measured using a salivary cortisol enzyme immunoassay (EIA) kit (Salimetrics, Philadelphia, PA, USA). The intra-assay variability was 4.85%, and inter-assay variabilities for high and low controls were 4.0 and 9.16%, respectively (mean of nine plates). In our previous study, we reported the stability of this method of HC measurement regardless of storage duration [57].

### Behavioral monitoring

Aggressive interactions were monitored using daily behavior monitoring sheets developed for this study. Aggressive interactions were defined as behaviors that included chasing, hitting, biting, kicking, and charging displays directed toward group members; individuals who were targets of such behavior showed screaming, escaping, or counterattacking behaviors(6). Each day, keepers or researchers in charge of taking care of the chimpanzees recorded the names of the individuals who initiated, and were the targets of, aggression when they observed any such behaviors during their husbandry routine. They also recorded whether the aggressive interactions resulted in injury to any individual involved. Because this is a simple way of behavior monitoring, we checked the validity of behavior monitoring sheets by comparing the sociogram generated by these sheets with that generated by direct observation. For that purpose, YY collected the behavioral data of 11 male chimpanzees living in two social groups for 6 months, in total, in 2014 and 2015 (from June to July 2014 and from December 2014 to March 2015). The daily checks of the behaviors using the daily behavior monitoring sheets by researchers and keepers continued during the same 6-month period. YY completed 230.5 hours of observation and collected data on aggressive interactions. Direct observations were made in the two adjacent groups of chimpanzees between 11 am and 3 pm. During the observation, YY collected data on aggressive interactions using all-occurrence sampling methods. YY conducted 30 min of focal observation in a randomly assigned order and recorded behaviors (e.g., foraging, resting, and grooming, which were not used for this study). During the observations, YY recorded all of the aggressive interactions that occurred in the groups. YY stopped making focal observations and recorded aggressive interactions when a scream was heard or when there were any other signs of aggression. We applied the same definition of aggressive interactions given above. We created sociograms based on these two types of observation. Pearson’s correlation tests were performed using UCINET [72]. The two observation methods yielded significant positive correlations in the two groups (r = 0.496–0.623, p < 0.01). We also tested the inter-rater reliability among the observers using video clips and found more than 90% agreement with the behaviors coded by the first author.

### Study 1: Monitoring HC level before and after relocation

For the first experiment, we collected hair from eight chimpanzees moved from the GARI to KS at the end of January 2013 (Table 1). They were divided into two groups: the main group, consisting of six chimpanzees (Group A), and another group, with a mother–offspring pair (Group B). We collected hair from these chimpanzees twice before relocation (August and December 2012) and seven times (first year: April, June, September, December, 2013 and March, 2014; second year: September 2014 and March 2015) after relocation. We collected hair more frequently (once every three months) for the first year after their move to the new environment. In September 2015, two individuals, Loi and Tsubaki, were moved to another zoo in Japan as part of a breeding program. Therefore, we collected hair from these two chimpanzees eight times, and we collected hair from the other six individuals nine times. We analyzed HC levels as described in the previous section. We divided the proximal and distal segments of the hair samples by cutting the hairs into two parts at the middle and processed these separately whenever possible. For comparison, we collected hair from 24 chimpanzees that remained in KS during this period (the control group); hair was collected seven times (once between July and December, 2012; in June, September, and December, 2013; in March and September, 2014; and in March, 2015).

**Table 1:** Profile of chimpanzees in Study 1.

Name	Year born	Sex	Rearing history	Group
Loi	1995	M	Artificial (late)	A
Zamba	1995	M	Artificial (late)	A
Mizuki	1996	F	Artificial (early)	A
Tsubaki	1996	F	Artificial (late)	A
Misaki	1999	F	Artificial (late)	B
Natsuki	2005	F	Mother-reared	A
Iroha	2008	F	Mother-reared	A
Hatsuka	2008	F	Artificial (early)	B

Aggressive interactions were recorded using behavior monitoring sheets from January 2013 to March 2014 by keepers and researchers during their husbandry routine. We categorized aggressive interactions into two categories: injurious (resulting in injury) and moderate (without injury).

There were various differences between the two institutions (GARI and KS) with respect to their physical environments and husbandry routines. For example, the GARI facility consisted of a large outdoor compound covering approximately 7,400 m^2^ in area and containing natural vegetation, enrichment items, and a 13-m high climbing structure attached to indoor rooms [73]. For the first 9 months after moving to KS, the eight chimpanzees (January 2012 to October 2013) lived in a facility consisting of two outdoor cages, each of which was approximately 100 m^2^ in area and 5.6 m in height, and attached to indoor rooms. Both the indoor and outdoor cages were enriched with items such as substrate materials, branches, fire hoses, and feeders. In October 2013, the chimpanzees were moved into a different facility within KS. This second facility consisted of three connected outdoor cages of approximately 70, 80, and 150 m^2^ in area and 5.4 m in height, containing natural vegetation and climbing frames attached to indoor rooms. The two facilities also differed with respect to food varieties and keepers. For more information regarding these two institutions, please see [12,74].

### Study 2: Relationship between HC level and environmental and individual factors

The subjects were 58 chimpanzees living in KS. We collected hair samples from the arm four times from 56 individuals and three times from two individuals between June 2013 and April 2014. We checked the effects of age, sex, relocation status, group type, rearing history and aggressive interactions on the average HC levels. The chimpanzees were divided according to relocation status: eight chimpanzees had been relocated (relocated from another institution as a group), seven chimpanzees were immigrants (relocated from other institutions and integrated into new social groups within 5 years), and 44 chimpanzees were residents (housed at KS for more than 5 years). Rearing history included four categories of chimpanzees: wild-born, mother-reared in captivity, and artificially reared (divided into early- or late-separation according to the timing of maternal separation). These categories are listed in Table 2. We used the rate of aggressive interactions calculated from behavior monitoring sheets recorded between April 2013 and March 2014 by keepers and researchers during their husbandry routine.

**Table 2:** Categories of individuals. We divided the artificially reared chimpanzees into two categories: early-deprived and late-deprived, as determined by median number of days of maternal separation.

Category	Definition	No. of males	No. of females
Group type			
All-male group	Group consists only of males	28	0
Mixed-sex group	Group consists of male(s) and females	7	23
Relocation status			
Relocation group	Relocated from another institution as a group	2	6
Immigrant group	Relocated from other institutions and integrated into new social groups within 5 years	5	2
Resident group	Housed at KS for more than 5 years	28	15
Rearing history			
Mother-reared	Reared by their biological mothers until weaning	7	2
Early-deprived	Separated from their biological mothers before 205 days and reared by humans	10	7
Late-deprived	Separated from their biological mothers after 333 days and reared by humans	5	4
Wild-born	Born in the wild	12	10
Unknown		1	0

### Statistical analysis

We used Generalized Linear Model (GLM) and Generalized Linear Mixed Model (GLMM) to analyze the results. For Study 1, we used GLMM to investigate the effects of relocation on eight chimpanzees. To this end, we used the function ‘glmer’ with a gamma distribution and the identity link function. We treated sampling year (three categories: before relocation, first year after relocation, and second year after relocation) as an explanatory variable and individual ID as a random factor. Due to convergence errors, we used changes in the HC level from 2012 as a response variable for constructing the model (the HC level for each year was divided by the HC level in 2012). We first constructed the model using sampling year, relocation-status group, and their interaction. Then, we separately analyzed changes in HC levels for each relocation-status group, and we compared Akaike Information Criteria (AIC) between models with and without the factor of sampling year as we found the interaction effects. Furthermore, to check the differences in the HC levels between two relocation-status groups for each sampling year, we constructed a model with relocation status group for each year and compared the AIC between models with and without the relocation-status group factor. For Study 2, we used the glm function with a gamma distribution and the linear function (identity) to examine the relationship between average HC levels and environmental and individual factors. We first constructed models including age, sex, relocation status, group type, rearing history, and aggressive interactions as explanatory variables. The rate of receiving and initiating aggression was calculated by summing the number of episodes involving the receiving of or initiation of aggression over an entire year; the rate was standardized for each social group. Standardization involved subtracting the average rate of receiving/initiating aggression within each social group from the individual rate of receiving/initiating aggression, and then dividing this number by the standard deviation for each group. As initiating aggression and receiving aggression can be related to each other, we constructed models including one of the factors to separately analyze these two factors. We also constructed models for each sex, because group type and aggressive interactions were confounded with sex. We included age, relocation status, group type, rearing history, and aggressive interactions as explanatory variables. In total, we constructed six models from sex (3 categories: all, male, and female)* and aggressive interactions (2 categories: receive and initiate aggression) and conducted a model simplification process [75]. We compared AIC among models comprised of various combinations of the aforementioned factors to identify the models that produced the lowest AIC value. We considered models with ΔAIC (difference in AIC from the smallest AIC models) < 2 as valid models [76]. We also used Spearman’s rank correlation test to examine the relationship between HC level and the number of months after relocation in immigrant chimpanzees. The alpha level was set at 0.05. R 3.1.2 was used for the statistical analysis [77].

### Ethics statement

This study was carried out in accordance with the recommendations in the "Guide for the Care and Use of Laboratory Primates 3rd Edition" of the Primate Research Institute, Kyoto University and the "Guide for Animal Research Ethics" of the Wildlife Research Center, Kyoto University. The samples were collected by people who were familiar with each of the chimpanzee subjects. No invasive procedures, such as anesthesia, were used in this study, and the care of chimpanzees is described in the Method section. The study protocol was approved by the institutional committee of the Wildlife Research Center (No. WRC-2014KS001A).

## Results

### Study 1–1: Changes in HC level before and after relocation

**Fig 1:**
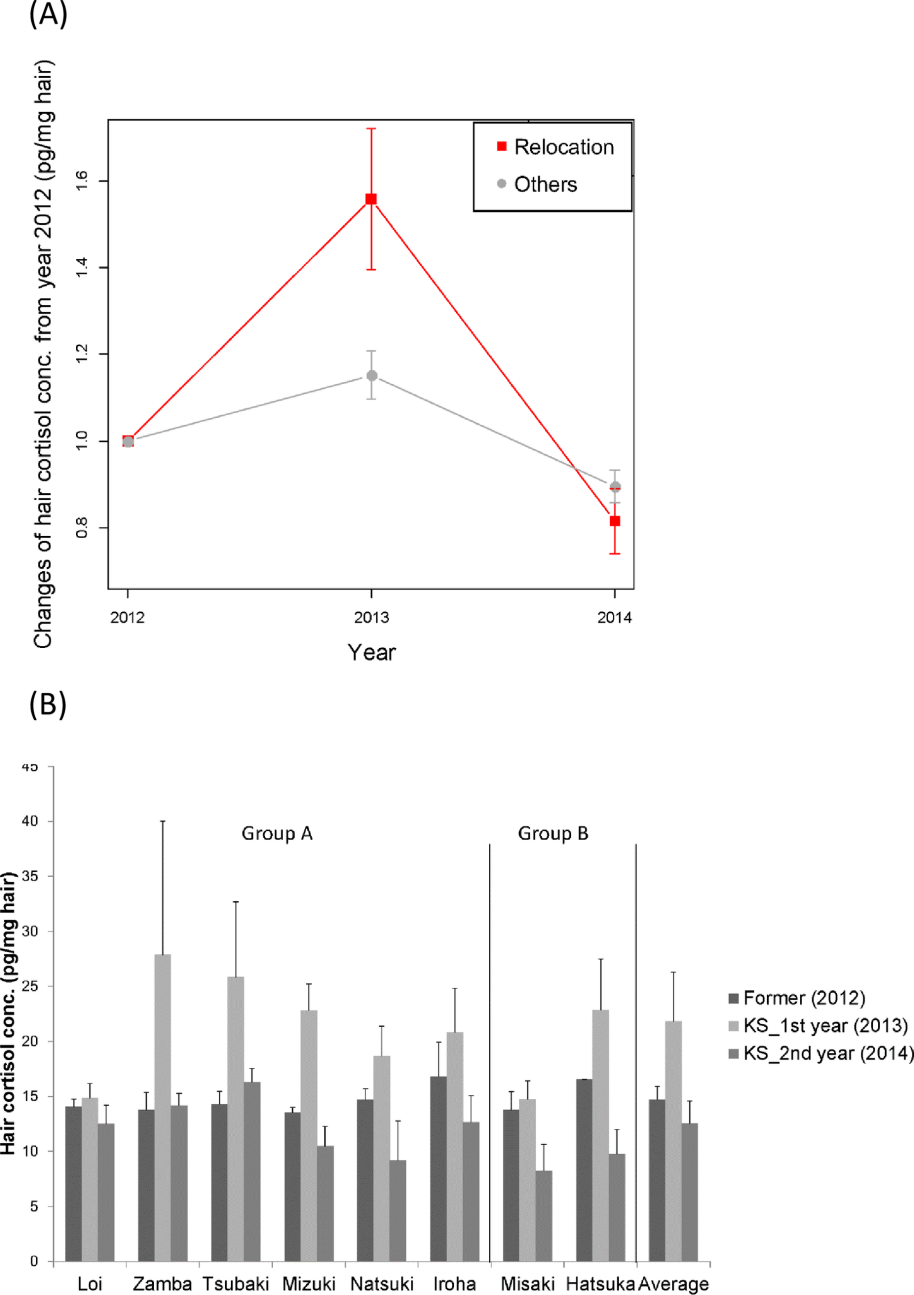
HC levels before and after relocation. (A) Changes in average HC levels of relocation group chimpanzees (Relocation) and control group chimpanzees (Others). (B) individual HC levels.

The model with sampling year, relocation-status group, and their interaction resulted in a lower AIC than the model without the interaction (Table 3: AIC with interaction -54.2; AIC without interaction -34.0). As we found interaction effects, we analyzed the effects of sampling year for each group separately. The model with sampling year produced a lower AIC than the null model for both the relocation group (AIC with sampling year 1.3, AIC without sampling year 27.6) and the control group (AIC with sampling year –50.2, AIC without sampling year -23.7.). Overall, HC level increased in the first year (2013) after relocation and decreased again in the second year (2014) after relocation compared with that in the former institution (2012) (Fig 1A; Year 2012: mean = 14.5 pg/mg hair; Year 2013: mean = 22.2 pg / mg hair, est. = 0.496, SE = 0.104, t = 4.77, p < 0.001; Year 2014: mean = 11.7 pg / mg hair, est. = -0.227, SE = 0.0716, t = -3.17, p = 0.00153). The control group also showed changes in the HC levels (Fig 1A; Year 2012: mean = 21.7 pg / mg hair; Year 2013: mean = 23.1 pg / mg hair, est. = 0.103, SE = 0.0427, t = 2.39, p = 0.0164; Year 2014: mean = 18.5 pg / mg hair, est. = -0.144, SE = 0.0373, t = -3.86, p < 0.001). Although both the relocation and control groups showed changes in HC levels, the HC levels of the two groups differed significantly for 2013 (AIC with relocation-status group 18.4; AIC without relocation-status group 25.0; est. = -0.406, SE = 0.150, t = -2.70, p = 0.0112), whereas it was not different in 2014 (AIC with relocation-status group 14.1; AIC without relocation-status group 15.1; est. = -0.109, SE = 0.068, t = -1.60, p = 0.113) Together with the aforementioned interaction effects, this result indicates that the range of fluctuation was wider in relocation-group chimpanzees. Six of eight individuals in the relocation group showed a similar tendency, but two individuals (one of whom was the alpha male of the group) did not show any change in HC level over the 3-year period (Fig 1B).

**Table 3:** Parameter estimates of the model with interaction.

Factor	Estimate	SE	T	P
Sampling year				
2013	0.495	0.0928	5.34	< 0.001**
2014	-0.227	0.0642	-3.55	< 0.001**
Group				
KS	-0.00138	0.0907	-0.015	0.988
Interaction				
2013 × Group KS	-0.393	0.0103	-3.83	<0.001**
2014 × Group KS	0.0838	0.0749	1.12	0.263

### Study 1–2: Changes in aggressive interactions after relocation

**Fig 2:**
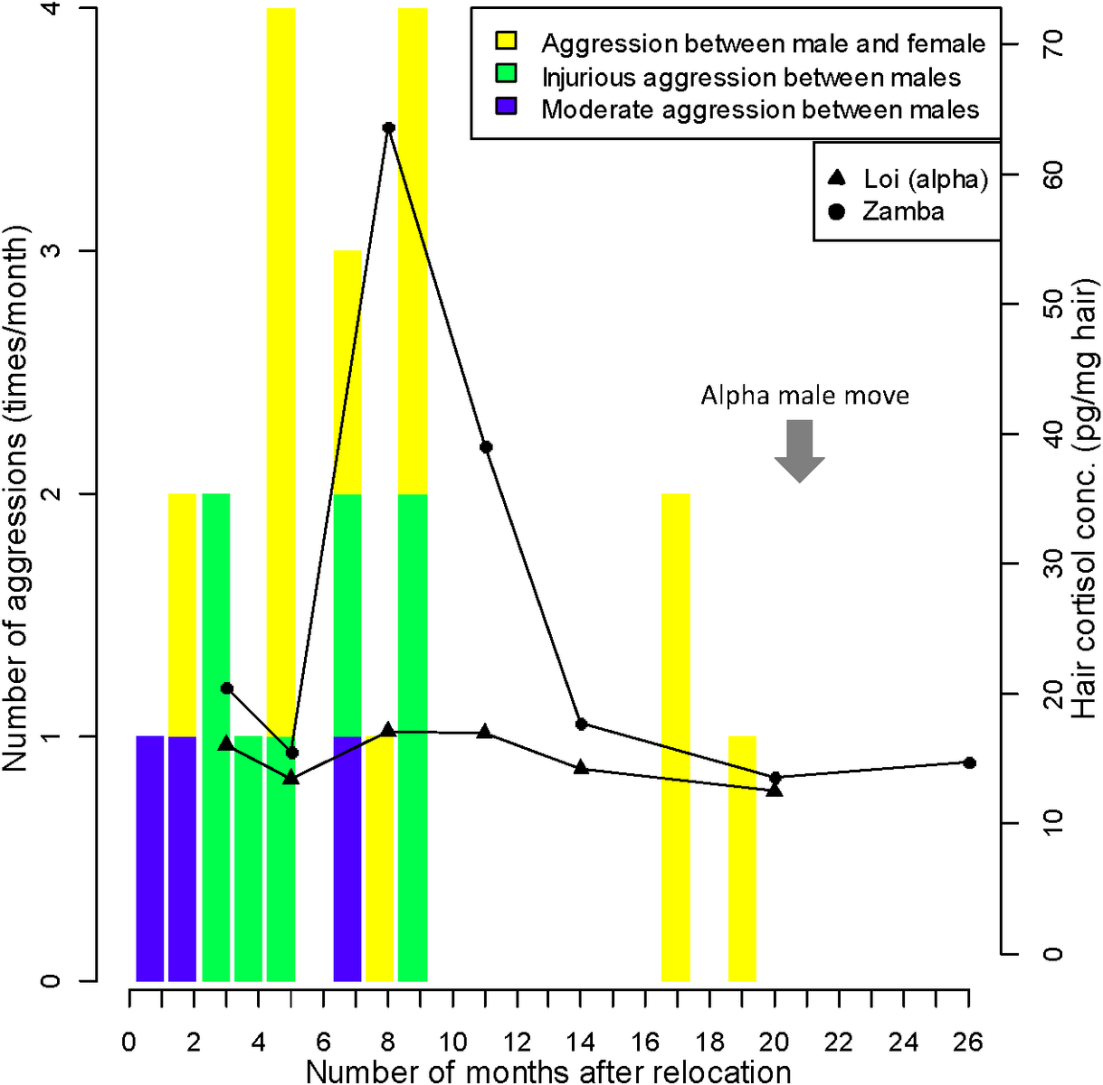
Changes in aggressive behaviors by an alpha male and HC levels of two males after relocation. Bar plots indicate the number of aggressive encounters initiated by an alpha male toward group members. Line charts indicate changes in HC concentration in this male and one other after relocation. The alpha male (Loi) moved to another institution in the 20^th^ month.

As we found clear individual differences in the reaction toward relocation, even among males, we checked the changes in aggressive interactions and HC levels. Aggressive interactions between males were recorded sporadically during the early period after relocation. Most of the aggressive interactions were directed by the alpha male toward a subordinate male, and these sometimes resulted in injury. Aggression between males ceased after November 2013 (Fig 2). The strongest cortisol response of the subordinate male was observed in the hair sample obtained in September 2013, which corresponds to the heightened period of aggression.

### Study 2: Relationship between HC level and individual and environmental factors

**Fig 3:**
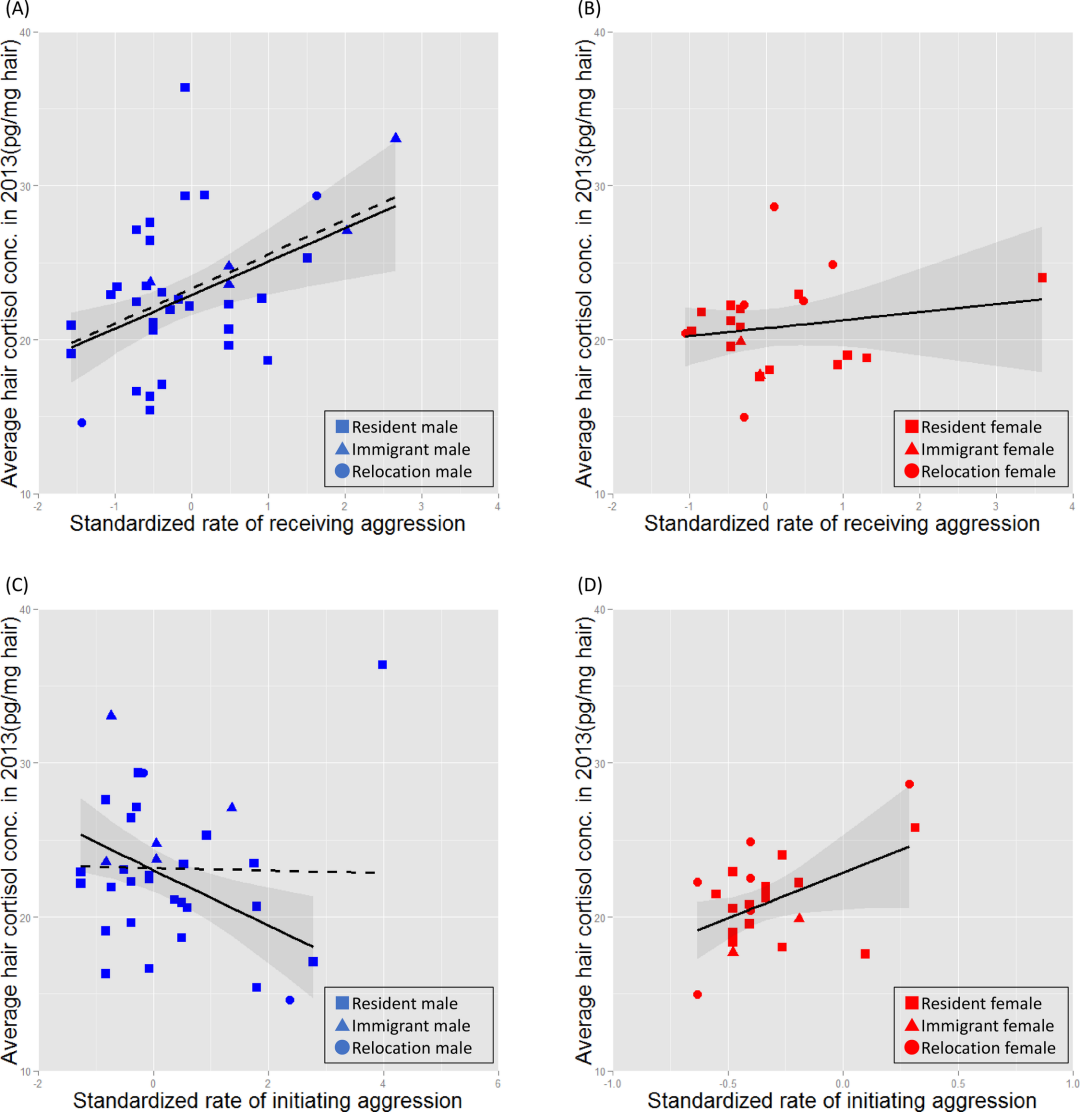
**Relationships between aggressive behaviors and HC levels: relationship between HC levels and receiving aggression (A,B) and between HC levels and initiating aggression (C,D).** The relationship between HC and aggression in males (A and C) and in females (B and D). The solid line in A and C indicates the fitted line generated from the male data with one outlier male removed. The dashed line indicates the fitted line including all male subjects.

When we compared the models including both sexes and the rate of receiving aggression, we found that sex, rate of receiving aggression, group type, and rearing history significantly influenced average HC level (Fig 3 and Table 4). In terms of the models that included initiating aggression, we excluded one obvious outlier individual shown in Fig 3C. There were three competing models, with ΔAIC < 2 (Table 4). All models contained the factor of sex and group type, and two models contained the factors of initiating aggression, and rearing history. According to the parameter estimates of the best-fit models (Table 5), males showed higher levels of HC concentration than females. In terms of rearing history, late-deprived individuals showed lower levels of HC than other groups of chimpanzees. Receiving aggression and initiating aggression followed opposite trends, as individuals receiving higher levels of aggression had higher levels of HC, and those initiating higher levels of aggression had lower levels. Relocation status and age were excluded from the models. Spearman’s rank correlation test was performed to examine the relationship between number of months after relocation and HC level in immigrant chimpanzees, but no significant relationship was identified (Spearman’s rank correlation, rho = -0.0561, n = 7, p = 0.905).

**Table 4:** AIC tables of the models to explain variation in the average HC levels of KS chimpanzees in 2013.

	Model with receiving aggression		Model with initiating aggression	
Male	Factors	AIC	Factors	AIC
Best-fit model	Receiving aggression + Rearing history+ Group Type	188.27	Initiating aggression + Rearing history + Group Type + Relocation status	184.7
			Initiating aggression + Rearing history + Group Type + Relocation status + Age	185.11
Female				
Best-fit model	Null	118.75	Initiating aggression	113.75
	Relocation status	120.27	Initiating aggression + Relocation status	114.06
Both sexes				
Best-fit model	Sex + Receiving aggression + Rearing history + Group Type	317.59	Sex + Initiating aggression + Group Type	313.55
			Sex + Initiating aggression + Group Type + Rearing history	314.4
			Sex + Rearing history + Group Type	315.37

**Table 5:** Parameter estimates from the models used to explain variation in the average HC levels of KS chimpanzees in 2013.

Male	Model with receiving aggression					Male	Model with initiating aggression			
Factor	Mean hair cortisol (pg/mg±SD)	Estimate	SE	T	P	Factor	Estimate	SE	T	P
(Intercept)		24.05	1.10	22.0	<0.001**	(Intercept)	22.3	3.59	6.19	<0.001 **
Group type						Group type				
All-male	22.3±3.89					All-male				
Mixed-sex (male)	26.6±6.85	3.98	1.10	2.58	0.0155**	Mixed-sex (male)	4.85	2.12	2.29	0.0308 *
Rearing						Rearing				
Artificial (Early)	24.7±3.03					Artificial (Early)				
Artificial (Late)	20.7±5.92	-6.31	1.64	-3.85	<0.001**	Artificial (Late)	-4.43	1.93	-2.294	0.0304 *
Mother-reared	26.1±6.03	0.85	1.82	0.468	0.643	Mother-reared	-1.78	1.84	-0.966	0.343
Wild-born	22.3±3.49	-1.75	1.42	-1.23	0.227	Wild-born	-3.14	1.4704	-2.134	0.0429
Aggressive interaction						Aggressive interaction				
Receive		2.27	0.625	3.63	0.0013**	Initiate	-1.56	0.596	-2.62	0.0148 *
Relocation status						Relocation status				
Relocation	21.9±10.4					Relocation				
Immigrant	26.4±3.96					Immigrant	5.69	3.91	1.46	0.157
Resident	23.2±4.82					Resident	1.61	3.29	0.491	0.628
Female						Female				
(Intercept)		21	0.634	33.2	<0.001 **	(Intercept)	23.1	1.01	22.8	<0.001**
Null						Aggressive interaction				
						Initiate	6.35	2.44	2.6	0.0166*
Both sexes						Both sexes				
(Intercept)		17.9	1.96	9.17	<0.001**	(Intercept)	17.5	1.99	8.78	<0.001 **
Sex						Sex				
Female	21.0±3.04					Female				
Male	23.16±4.82	5.02	1.76	2.86	0.00622**	Male	4.87	1.91	2.55	0.0137 *
Group type						Group type				
All-male	22.3±3.89					All-male				
Mixed-sex (male)	26.6±6.85	3.71	1.74	2.13	0.0381 *	Mixed-sex (male)	3.07	1.81	1.69	0.0965 +
Mixed-sex (female)	21.0±3.04					Mixed-sex (female)				
Rearing										
Artificial (Early)	22.9±3.34									
Artificial (Late)	20.1±5.50	-3.02	1.39	-2.18	0.0339 *					
Mother-reared	25.1±5.63	1.3	1.63	0.8	0.428					
Wild-born	21.8±3.10	-0.91	1.16	-0.795	0.431					
Aggressive interaction						Aggressive interaction				
Receive		1.53	0.53	2.88	0.00585 **	Initiate	-1.56	0.599	-2.61	0.0119 *

As sex differences were observed in terms of average HC level, aggressive interactions, and group type, we constructed and selected models for each sex. The models for the male data with explanatory variables that were similar to those in models for both sexes (group type, rearing history, and aggressive interactions) produced the lowest AIC. Although the factor relocation status remained in the final model with initiating aggression, relatively smaller parameter estimates, with relatively high standard errors, were observed for this factor compared with the other variables. However, only initiating aggression remained in the final model for female data. In addition, according to the parameter estimates, females with a higher level of aggression had higher HC concentrations, which was opposite to the pattern for males.

## Discussion

This study showed that HC level increased during the first year after relocation to the new environment and that it decreased during the second year. Although chimpanzees in both the control and relocation groups showed changes in HC levels, the most pronounced change in HC was observed in the relocation-group chimpanzees, which might suggest the operation of relocation stress in the first year and acclimation effects in the second year after relocation, as predicted. As there were many differences between the two institutions, it is difficult to determine the specific factors influencing the changes in HC and aggressive behaviors. Although we did not find clear sex differences, social factors represent one possible type of mediator, given that there were individual differences in reactions to relocation. The alpha male did not show changes in HC levels, whereas the other male, who received aggressive interactions, showed the highest increase in HC levels. The timing of the increase in HC levels in the subordinate male corresponded to the periods of heightened aggression. Thus, not only relocation but also social factors might influence changes in HC concentration.

Study 2 revealed that the standardized rate of receiving and initiating aggression had a significant effect on HC levels, whereas relocation status alone did not. Indeed, data from newly arrived chimpanzees (including both immigrant and relocation-group chimpanzees) varied widely and were not related to the number of months since relocation. Combining studies 1 and 2, the most important factor affecting long-term stress levels after moving to a new environment might be whether the new chimpanzees received aggression.

However, there were sex differences, as males showed higher HC concentrations than females, and the association with aggressive interactions was stronger in males. The direction of the relationship between HC and the rate of initiating aggression was opposite in males and females, although this should be treated with caution considering the limited range of variations in aggressiveness among females. These associations between aggressive behaviors and sex were consistent with our previous study in male chimpanzees [54] and previous findings in female ring-tailed lemurs [78,79] showing that higher ranked females had higher fecal GC levels than did lower ranked individuals, and that GC level was positively associated with the rate of initiating aggression [78]. Males and females may be affected by their social environment in different ways; this tendency should be considered when planning the relocation of captive chimpanzees. In the wild, female chimpanzees migrate from their natal groups after puberty, whereas males generally remain in their original groups [4]. Although the small sample size of female immigrant chimpanzees limits our discussion of relocation and sex differences, two female immigrant chimpanzees showed low HC levels and low rates of receiving aggression. Kahlenberg et al. [17] reported that immigrant females among the wild chimpanzees of Kibale National Park received higher levels of aggression from female residents and exhibited higher urinary cortisol levels than did resident females. They also reported that immigrant chimpanzees received less aggression in the presence of male chimpanzees. In the current study, males were present most of time due to the spatial limitations of the environment, which may have influenced our results.

In this study, the relationship between receiving and initiating aggression and HC followed opposite patterns among males, although the previous study in wild chimpanzees reported that single aggressive interactions in both directions can increase urinary cortisol metabolite levels [80]. These differences might be related to the fact that HC reflects long-term rather than short-term stress and to the possibility that initiating aggression might increase cortisol in the short-term but not the long-term. Muller and Wrangham [81] reported that dominant individuals in Kibale National Park showed higher levels of fecal GC metabolites. In their study, dominance rank was decided based on a model that took into consideration the number of opponents that an individual successfully defeated. The present study found patterns that were in the opposite direction to those observed in their study. We also used the relative level of aggressiveness within the group as a measure, although methodological differences exist between the two studies. However, variations in results have often been reported in previous research involving rats, human and non-human primates; the possible explanations offered were social stability, physical environment, and controllability [18,26,82]. In fact, the alpha male who showed the highest level of aggression in a mixed-sex group also showed the highest average HC concentration in the present study (one outlier male in Fig 3C). Although the aforementioned factors probably contributed to the variations in this study, the current study did not have a large enough sample size to elucidate the factors underlying this type of variation. As long-term elevation of cortisol levels may be detrimental to health and behavior [26], such high-HC individuals should be closely monitored; a detailed study of the factors affecting stress levels is also important for the maintenance of the good health of such exceptionally high-cortisol individuals.

There was no evidence that an all-male grouping leads to higher levels of HC. This is an important result, because all-male groups do not exist in wild chimpanzees, which is unlike the case for wild gorillas, which sometimes form groups of this kind [66,83]. Due to the aggressiveness of male chimpanzees, multi-male and multi-female social groupings are often difficult to form [2,13], and social groups with one male and several females are more widespread. As a result, surplus males emerge and, in the worst cases, can be isolated. All-male groups can be an alternative to reducing the number of isolated males in captivity without imposing excessive stress, as long as we minimize the incidents of escalated aggression. However, there were only a limited number of social groups in this study, and we should note that the keepers were careful to avoid escalated aggression, monitoring and adjusting the membership of groups, especially when new males were introduced. Future studies of all-male groups of captive chimpanzees should use larger samples and include other welfare-related parameters, such as behaviors and other physiological variables.

The results related to rearing history were unexpected, because late-deprived individuals showed lower HC concentrations than wild-born, early-deprived, and mother-reared individuals, especially among males. Dettmer et al. [63] reported that rhesus macaques who were reared by their mother showed less pronounced responses to relocation and that the HC levels of mother-reared individuals were lower than those of peer-reared and nursery-reared individuals when assessed at 6 and 18 months. However, opposite patterns were also reported in many earlier studies on the effects of maternal separation [68]. Studies of rodents and primates indicated that early adverse experiences decrease basal cortisol levels (hypocortisolism) later in life [67,84,85]. For example, Feng et al. [86] reported that rhesus macaques who experienced maternal separation in early life had lower basal HC levels and delays in their response to stressors (capture and restraint), assessed by plasma cortisol responses, compared with mother-reared conspecifics. Researchers have reviewed the effects of maternal separation on infant development and discussed factors, such as species differences, type of stress, sex, timing of maternal separation and differences in the details of "maternal separation" as potential explanations for these inconsistent results [87]. This study showed that the sex and timing of maternal separation had different effects on basal cortisol levels. In terms of the timing of maternal separation, hypocortisolism was observed only in late-deprived individuals and not in early-deprived individuals. It is difficult to attribute the results to maternal separation based only on alterations in the HPA axis. Indeed, the fact that these two groups of chimpanzees might differ in their social abilities may have influenced the results. For example, many late-deprived individuals in this study occupied higher-ranking positions, although there was no direct relationship between rearing history and aggressive behaviors. Reimers et al. [62] found that late-deprived resocialized captive chimpanzees showed lower levels of fecal GC metabolites than early-deprived ones. At the same time, the late-deprived individuals were more highly ranked than the early-deprived individuals in their study. Thus, rearing history might influence stress response partly because rearing history can be related to how individuals form social relationships. However, there is an alternative hypothesis for explaining the results. As some studies have reported the possible salutary effects of later experiences on the sequelae of maternal separation[88], social experiences later in life might buffer the effects of early-life experiences. Unlike subjects in the earlier experimental studies, all subjects in this study spent many years in social environments. Thus, the current environment of the animals may have had more pronounced effects on the HC levels of the chimpanzees than the early one. In the current study, we could not dissociate the contribution of early rearing conditions from that of current social environment to HC levels, partly due to the fact that the detailed rearing histories of wild-born individuals and some captive-born individuals were unclear. Additional studies including a larger sample are needed to understand how the combination of early rearing conditions and current environment affects the stress response of animals.

Age was not a significant contributor to either average HC level. This was consistent with our previous study [54], although the age range was expanded to between 5 and 44. Studies have found age-related declines in HC levels from infancy in rhesus macaques, baboons, and vervet monkeys [45,63,89]. The fact that our study did not include infants might be why we did not find any association with age.

There were some limitations to this study, pertaining to our understanding of the specific factors contributing to HC levels. Although the daily behavior monitoring sheets provided valuable information, we included aggressive interactions as only a social index due to methodological limitations, as our methodology rendered it impossible to record quantitatively frequent but less evident social behaviors, such as social grooming. There is some evidence that affiliative social interaction, such as grooming, can be related to stress alleviation in primate species [90–93]. Furthermore, the size of enclosures was reduced after the move to KS; such a change may have influenced the social behavior of the chimpanzees. The intense aggression by the alpha male to subordinate males ceased 9 months after relocation, which coincided with the timing of changes to the facility inside the new institution; however, this did not result in large differences in the size of enclosures. The decrease in aggression may have been due to the simple passage of time, although we cannot rule out the possibility that the intense aggression was influenced by properties of the physical environment. If aggressive interactions strongly influence the long-term stress level after relocation and if it, in turn, is influenced by physical environment, then it may be possible to alleviate long-term stress by modifying certain properties of the physical environment. Future research investigating other social parameters is needed to achieve a detailed understanding of the effects of the social environment on chimpanzee welfare and to evaluate the role of physical environmental parameters in the alleviation of the stress experienced by these chimpanzees.

In conclusion, relocation can affect long-term stress, but the issue of whether individuals receive aggression might be a more important contributor to long-term stress than relocation alone. Group type, rearing history, and sex were also related to HC levels in captive chimpanzees. Male and female chimpanzees might be affected by the social environment differently, which might be related to social characteristics in the wild. These sex differences should be considered more carefully when planning the relocation and formation of captive social groups. This study further strengthened the usefulness of HC analysis as a means of monitoring long-term stress, especially in the context of social management. Future studies should focus more on the consequences of higher HC levels in terms of other welfare parameters to ensure the physical and mental health of chimpanzees and to contribute to efforts to alleviate their stress.

## Supporting Information

S1 Table Changes in hair cortisol levels before and after the relocation. Data used for investigating the effects of relocation on hair cortisol levels (Study 1). Changes of HC levels from the year 2012 were used. Age information was at year 2013.
(DOCX)

S2 Table Average hair cortisol levels in 2013 and relevant information. Data used for investigating the effects of individual and environmental factors affecting hair cortisol levels (Study 2).
(DOCX)
