# Supplementary material for: Effects of Relocation and Individual and Environmental Factors on the Long-Term Stress Levels in Captive Chimpanzees (Pan troglodytes): Monitoring Hair Cortisol and Behaviors
Source: PLoS One. 2016 Jul 27;11(7):e0160029. doi: 10.1371/journal.pone.0160029 (PMC4963107; doi:10.1371/journal.pone.0160029)
Supplement: S1 Table — Data used for investigating the effects of relocation on hair cortisol levels (Study 1). Changes of HC levels from the year 2012 were used. Age information was at year 2013. (DOCX) [file pone.0160029.s001.docx]

**S1 Table. Changes in hair cortisol levels before and after the relocation.**

Data used for investigating the effects of relocation on hair cortisol levels (Study 1). Changes of HC levels from the year 2012 were used. Age information was at year 2013.

| Relocation status | Sex | Age | Y2012 | Y2013 | Y2014 |
| --- | --- | --- | --- | --- | --- |
| Relocation | F | 5 | 1 | 1.345479 | 0.590281 |
| Relocation | F | 5 | 1 | 1.13102 | 0.63506 |
| Relocation | F | 8 | 1 | 1.496558 | 0.675205 |
| Relocation | F | 14 | 1 | 1.23984 | 0.682144 |
| Relocation | F | 17 | 1 | 1.902935 | 0.80027 |
| Relocation | F | 17 | 1 | 1.851726 | 1.052842 |
| Relocation | M | 18 | 1 | 1.092389 | 0.934557 |
| Relocation | M | 18 | 1 | 2.401549 | 1.158982 |
| Control | M | 14 | 1 | 0.691428 | 0.673944 |
| Control | M | 16 | 1 | 1.160553 | 0.604225 |
| Control | M | 18 | 1 | 1.57216 | 1.098965 |
| Control | M | 18 | 1 | 0.849239 | 0.76702 |
| Control | M | 21 | 1 | 1.069951 | 0.746047 |
| Control | M | 23 | 1 | 1.241483 | 0.781687 |
| Control | M | 23 | 1 | 1.100984 | 0.77059 |
| Control | M | 23 | 1 | 0.90183 | 0.846468 |
| Control | M | 23 | 1 | 1.074725 | 1.049872 |
| Control | M | 23 | 1 | 1.01911 | 0.904283 |
| Control | F | 24 | 1 | 1.317211 | 0.967861 |
| Control | M | 24 | 1 | 1.069816 | 0.883103 |
| Control | M | 26 | 1 | 0.621648 | 0.510655 |
| Control | F | 27 | 1 | 1.870849 | 1.068482 |
| Control | M | 31 | 1 | 1.423617 | 1.068822 |
| Control | M | 31 | 1 | 1.182364 | 1.312297 |
| Control | M | 32 | 1 | 1.10835 | 0.937476 |
| Control | F | 33 | 1 | 1.419627 | 1.080105 |
| Control | M | 34 | 1 | 1.035608 | 0.799752 |
| Control | F | 35 | 1 | 0.988766 | 1.161548 |
| Control | F | 38 | 1 | 1.421478 | 0.878018 |
| Control | M | 38 | 1 | 1.101077 | 0.78634 |
| Control | F | 39 | 1 | 1.296539 | 0.980447 |
| Control | M | 42 | 1 | 1.106589 | 0.815463 |
