# Supplementary material for: Effects of Relocation and Individual and Environmental Factors on the Long-Term Stress Levels in Captive Chimpanzees (Pan troglodytes): Monitoring Hair Cortisol and Behaviors
Source: PLoS One. 2016 Jul 27;11(7):e0160029. doi: 10.1371/journal.pone.0160029 (PMC4963107; doi:10.1371/journal.pone.0160029)
Supplement: S2 Table — Data used for investigating the effects of individual and environmental factors affecting hair cortisol levels (Study 2). (DOCX) [file pone.0160029.s002.docx]

**S2 Table. Average hair cortisol levels in 2013 and relevant information.**

Data used for investigating the effects of individual and environmental factors affecting hair cortisol levels (Study 2).

| Group type | Relocation status | Sex | Rearing | Age | Abnormal behaviors | Initiating aggression | Receiving aggression | Hair cortisol concentration (pg/mg hair) |
| --- | --- | --- | --- | --- | --- | --- | --- | --- |
| Mixed-Sex | Relocation | F | Early | 5 | 4 | -0.6344813 | -0.2879145 | 22.25646 |
| Mixed-Sex | Relocation | F | Mother | 5 | 3 | -0.4037608 | 0.4798574 | 22.51437 |
| Mixed-Sex | Relocation | F | Mother | 8 | 1 | -0.4037608 | -1.0556864 | 20.41242 |
| All-Male | Resident | M | Mother | 14 | 2 | -0.0731095 | -0.7163971 | 22.43395 |
| Mixed-Sex | Relocation | F | Late | 15 | 2 | -0.6344813 | -0.2879145 | 14.98139 |
| Mixed-Sex | Resident | M | Mother | 17 | 1 | 0.5285834 | -0.9713937 | 23.43833 |
| Mixed-Sex | Relocation | F | Early | 17 | 6 | -0.4037608 | 0.8637434 | 24.86502 |
| Mixed-Sex | Relocation | M | Late | 18 | 1 | 2.364885 | -1.43957 | 14.61753 |
| Mixed-Sex | Relocation | F | Late | 18 | 4 | 0.288401 | 0.095971 | 28.62074 |
| Mixed-Sex | Resident | M | Early | 18 | 3 | -0.2627279 | -0.0827892 | 29.32078 |
| Mixed-Sex | Relocation | M | Late | 18 | 2 | -0.1730404 | 1.6315153 | 29.33393 |
| All-Male | Resident | M | Late | 19 | 5 | -0.3856309 | 0.4822538 | 19.63511 |
| All-Male | Resident | M | Early | 20 | 3 | -0.7310947 | -0.2811178 | 21.94037 |
| Mixed-Sex | Resident | F | Early | 21 | 10 | -0.47854 | 1.3135892 | 18.80268 |
| Mixed-Sex | Resident | F | Early | 22 | 10 | -0.4066026 | -0.3366762 | 20.80014 |
| All-Male | Resident | M | Mother | 22 | 5 | 0.4884658 | -1.5673248 | 20.92645 |
| All-Male | Resident | M | Mother | 22 | 3 | -1.2597275 | -1.0549301 | 22.92189 |
| Mixed-Sex | Resident | M | Mother | 23 | 4 | 1.7515191 | -0.5905632 | 23.45886 |
| All-Male | Immigrant | M | Early | 23 | 6 | 0.0514175 | 0.4822538 | 24.73212 |
| All-Male | Resident | M | Early | 23 | 3 | 0.9255141 | 1.507043 | 25.29051 |
| All-Male | Immigrant | M | Mother | 23 | 2 | -0.7310947 | 2.657017 | 33.04463 |
| All-Male | Resident | M | Late | 24 | 4 | -0.0731095 | -0.7163971 | 16.65232 |
| All-Male | Resident | M | Late | 24 | 3 | 2.77816 | -0.3899377 | 17.08194 |
| Mixed-Sex | Resident | F | Early | 24 | 5 | -0.47854 | 1.0597022 | 18.97423 |
| All-Male | Resident | M | Early | 24 | 3 | 1.7996107 | 0.4822538 | 20.68311 |
| Mixed-Sex | Resident | F | Early | 24 | 4 | -0.3346653 | -0.4636197 | 21.22356 |
| All-Male | Immigrant | M | Early | 24 | 2 | 1.3625624 | 2.0194377 | 27.0419 |
| Mixed-Sex | Resident | M | Mother | 24 | 3 | 3.9815782 | -0.0827892 | 36.37757 |
| All-Male | Resident | M | Early | 25 | 5 | -0.2924379 | -0.7163971 | 27.13055 |
| All-Male | Resident | M | Early | 26 | 7 | -0.8226792 | -0.5425355 | 27.61155 |
| All-Male | Resident | M | Early | 27 | 3 | 0.3655474 | -0.4987575 | 21.1194 |
| Mixed-Sex | Resident | F | Late | 28 | 3 | -0.47854 | 0.9327587 | 18.35771 |
| Mixed-Sex | Resident | F | Late | 31 | 3 | -0.3346653 | -0.3366762 | 21.99611 |
| All-Male | Resident | M | Early | 31 | 8 | -0.0731095 | -0.172298 | 22.59154 |
| All-Male | Resident | M | UK | 32 | 0 | 1.7996107 | -0.5425355 | 15.39879 |
| Mixed-Sex | Immigrant | F | Wild | 32 | 2 | -0.1907905 | -0.3366762 | 19.84986 |
| All-Male | Resident | M | Wild | 32 | 1 | -0.3856309 | 0.4822538 | 22.30234 |
| All-Male | Resident | M | Wild | 33 | 0 | -0.8226792 | -0.5425355 | 16.31608 |
| All-Male | Resident | M | Wild | 33 | 2 | -0.8226792 | -1.5673248 | 19.0821 |
| All-Male | Immigrant | M | Wild | 33 | 5 | -0.8226792 | 0.4822538 | 23.55175 |
| Mixed-Sex | Resident | F | Wild | 34 | 3 | -0.4066026 | -0.4636197 | 19.54354 |
| All-Male | Resident | M | Wild | 35 | 2 | -1.2597275 | -0.0301409 | 22.16633 |
| All-Male | Immigrant | M | Wild | 35 | 2 | 0.0514175 | -0.5425355 | 23.71445 |
| Mixed-Sex | Immigrant | F | Early | 36 | 11 | -0.47854 | -0.0827892 | 17.68655 |
| All-Male | Resident | M | Wild | 36 | 5 | -0.5117663 | -0.3899377 | 23.06031 |
| Mixed-Sex | Resident | F | Wild | 36 | 3 | 0.3127713 | -0.4636197 | 25.78054 |
| All-Male | Resident | M | Wild | 36 | 3 | -0.3856309 | -0.5425355 | 26.4254 |
| Mixed-Sex | Resident | F | Wild | 37 | 2 | -0.1907905 | -0.4636197 | 22.21486 |
| Mixed-Sex | Resident | F | Wild | 37 | 4 | -0.2627279 | 3.598572 | 24.00951 |
| Mixed-Sex | Resident | F | Wild | 38 | 2 | -0.3346653 | -0.8444502 | 21.78491 |
| Mixed-Sex | Resident | F | Wild | 39 | 3 | -0.2627279 | 0.0441543 | 18.02908 |
| All-Male | Resident | M | Wild | 40 | 5 | 0.5848758 | -0.4987575 | 20.60108 |
| All-Male | Resident | M | Wild | 40 | 3 | -0.0731095 | 0.9159001 | 22.67495 |
| Mixed-Sex | Resident | F | Wild | 40 | 2 | -0.47854 | 0.4249847 | 22.93253 |
| Mixed-Sex | Resident | F | Wild | 41 | 3 | 0.0969591 | -0.0827892 | 17.58489 |
| Mixed-Sex | Resident | F | Wild | 41 | 3 | -0.47854 | -0.9713937 | 20.53487 |
| Mixed-Sex | Resident | M | Wild | 43 | 3 | -0.2627279 | 0.1710977 | 29.37533 |
| All-Male | Resident | M | Wild | 44 | 1 | 0.4884658 | 0.9946484 | 18.63292 |
